# Common Reservoirs for *Penicillium marneffei* Infection in Humans and Rodents, China

**DOI:** 10.3201/eid1702.100718

**Published:** 2011-02

**Authors:** Cunwei Cao, Ling Liang, Wenjuan Wang, Hong Luo, Shaobiao Huang, Donghua Liu, Jianping Xu, Daniel A. Henk, Matthew C. Fisher

**Affiliations:** Author affiliations: The First Affiliated Hospital of Guangxi Medical University, Nanning, People’s Republic of China (C. Cao, L. Liang, W. Wang, H. Luo, D. Liu);; The Fourth Hospital of Nanning, Nanning (S. Huang);; McMaster University, Hamilton, Ontario, Canada (J. Xu);; Imperial College, London, UK (D.A. Henk, M.C. Fisher)

**Keywords:** Fungal infections, fungi, Penicillium marneffei, microsatellite typing, genotype, zoonosis, mycosis, sylvatic, China, research

## Abstract

Human penicilliosis marneffei is an emerging infectious disease caused by the fungus *Penicillium marneffei*. High prevalence of infection among bamboo rats of the genera *Rhizomys* and *Cannomys* suggest that these rodents are a key facet of the *P. marneffei* life cycle. We trapped bamboo rats during June 2004–July 2005 across Guangxi Province, China, and demonstrated 100% prevalence of infection. Multilocus genotypes show that *P. marneffei* isolates from humans are similar to those infecting rats and are in some cases identical. Comparison of our dataset with genotypes recovered from sites across Southeast Asia shows that the overriding component of genetic structure in *P. marneffei* is spatial, with humans containing a greater diversity of genotypes than rodents. Humans and bamboo rats are sampling an as-yet undiscovered common reservoir of infection, or bamboo rats are a vector for human infections by acting as amplifiers of infectious dispersal stages.

*Penicillium marneffei* is the only pathogenic species of *Penicillium* within this grouping of >270 species. This unique feature is due to the ability of *P. marneffei* to exhibit temperature-dependent dimorphic growth as an intracellular macrophage-associated fission yeast at 37°C. Before the HIV pandemic in Asia during the early 1990s, human penicilliosis was an exceedingly rare infection (*1*). Since then, however, this mycosis has become widely recognized as a co-infection in patients with HIV/AIDS, with an incidence that rivals that seen for *Cryptocococcus neoformans* and *Mycobacterium tuberculosis* (*1*). The organism is endemic across a narrow band of tropical Southeast Asia, with human- and rodent-associated infections occurring in northeast India, Thailand, the Guangxi region of China, Vietnam, Taiwan, and Hong Kong (*2**–**4*). Within these regions, *P. marneffei* has emerged as a major threat to public health; in Guangxi Province alone, ≈16% patients with AIDS are infected with the pathogen, and >100 new cases are reported from The First Affiliated Hospital of Guangxi Medical University per year (C. Cao, unpub. data). Although unproven, humans are assumed to become infected by inhaling aerosolized infectious conidia originating from thus far unidentified environmental sources (*1*).

Despite the growing cost of this infection to human health across this region, the reservoir for human infections remains enigmatic. One clue to the potential source of infection is that *P. marneffei* maintains a close association with rodent species, particularly bamboo rats. Across Thailand and Vietnam, *P. marneffei* is commonly recovered from species of *Cannomys* and *Rhizomys* bamboo rats, with prevalences of infection approaching 100%. The type isolate of the pathogen was identified from a sample from an infected *Rhizomys sinensis* rat in 1956 (*5*). The observation that *P. marneffei* is the only species of *Penicillium* to have evolved a pathogenic lifestyle strengthens the hypothesis that small mammals are an obligate phase in the life cycle of *P. marneffei*.

As with other dimorphic fungal pathogens that infect rodents, such as *Coccidioides* spp., infection in bamboo rats is assumed to lend a selective benefit by creating a nutrient-rich patch for sporulation and widespread aerosol-dispersal after the eventual death of the host (*6*). However, identifying penicilliosis infections in rodents as the ultimate sources of penicilliosis infections in humans requires, as a first step, a demonstration that the genotypes of sylvatic and human-associated isolates are similar or identical.

To this end, we ascertained the sylvatic prevalence of infection by trapping hoary bamboo rats (*Rhizomys pruinosis*) from across a region to which the infection is endemic, Guangxi Province in southern China, a region in which the observed case-rate for human penicilliosis marneffei is rapidly increasing (*1**,**2*). From these rodents, *P. marneffei* was isolated and genotyped by using a panel of highly polymorphic microsatellite loci. We also collected a panel of isolates from human infections across this region and then compared the distribution of genetic diversity within and between bamboo rats and humans across Guangxi and, more widely, Southeast Asia. These analyses were then used to identify the distribution of genetic diversity within and between hosts, identifying its major hierarchical components and identifying common genotypic features.

## Materials and Methods

### Study Area and Isolate Sources

All isolates were collected in Guangxi region of southern China on the southeastern corner of the Yunnan-Guizhou Plateau, situated from 20.54°N to 26.23°N and from 104.08°E to 112.04°E. This region borders Vietnam to the southwest and is surrounded by Guangdong, Guizhou, Yunnan, and Hunan Provinces in China. The region has a terraced topography sloping from the northwest to the southeast, with hilly land constituting 85% of its total area and plains constituting 15%. The region has a subtropical humid monsoon climate, with average daily temperatures of 16°C–23°C. The rainy season lasts from April until September, with an annual rainfall of 1,500 mm–2,000 mm.

**Figure 1:**
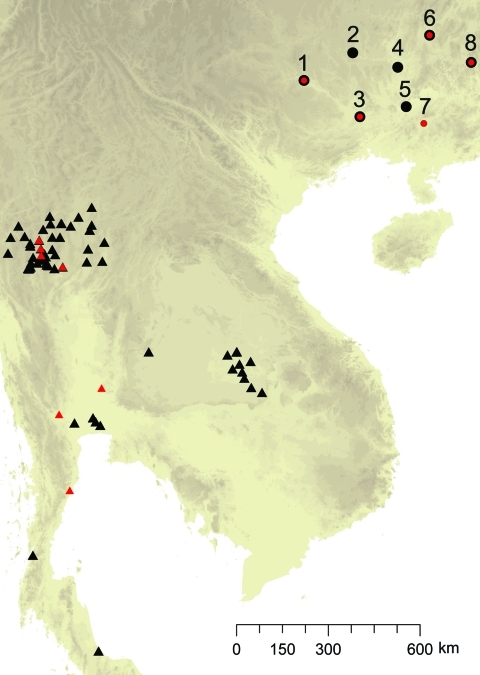
Spatial distribution of sampling sites for *Penicillium marneffei*, Guangxi Province, People’s Republic of China. 1, Bose; 2, Hechi; 3, Nanning; 4, Liuzhou; 5, Guigang; 6, Guiling; 7, Luchan; 8, Hezhou; Black signifies origin of human-associated isolates, and red signifies origin of bamboo rat–associated isolates; both types were found in some sites.

Farmers trapped 43 adult hoary bamboo rats (*R. pruinosus*) from 8 different districts across Guangxi Province. The 15 female and 28 male captured rats were euthanized and aseptically dissected as described (*7*). *P. marneffei* was recovered from the main organs of the rats (lungs, liver, and spleen) by injection onto Sabouraud dextrose agar and brain–heart infusion agar and cultured at 25°C and 37°C, respectively, for 3–4 weeks. Both media were supplemented with chloramphenicol (0.05 mg/mL). Species identification of *P. marneffei* was based on conversion of yeast to hyphae at 25°C, secretion of a characteristic bright red pigment, and morphologic identification of colonies and conidia formation. In addition to the rodent isolates, 40 isolates were collected from human patients (including 36 persons positive for HIV) across Guangxi Province. The clinical specimens included blood, skin biopsy samples, pus from subcutaneous abscesses, lymph node biopsy samples, and bronchoalveolar lavage pellets. The linear geographic distance among the sites ranged from 133 km to 503 km, with an average distance of 224 km (Table 1; Figure 1).

**Table 1:** Sampling information for *Penicillium marneffei* isolates, Guangxi Province, People’s Republic of China

Sampling site location	Coordinates	No. isolates	
		From rats	From humans
Liuzhou	24.275°E, 109.385°N	0	9
Hezhou	24.415° E, 111.547°N	10	5
Guigang	23.1159°E, 109.633°N	0	4
Hechi	24.71°E, 108.06°N	0	2
Nanning	22.815°E, 108.27°N	10	11
Guiling	25.219°E, 110.32°N	9	5
Bose	23.889°E, 106.626°N	9	4
Luchuan	22.62°E, 110.149°N	5	0

### Multilocus Microsatellite Typing of Isolates

DNA was extracted from 7-day-old cultures of each *P. marneffei* isolate as described (*8*). Six microsatellite-containing loci were chosen from the panel described by Fisher et al (*9*). These loci (PM5, PM6, PM19, PM22, and PM23) were selected because of their high discriminatory power within a previously genotyped cohort of isolates obtained from humans and bamboo rats in Thailand (*10*); the loci vary in the length of the microsatellite-containing repeat region. The 6 loci were amplified according to published PCR protocols (*9*). Subsequently, the PCR products were subjected to electrophoresis through a capillary sequencer with a POP6 gel and a ROX-500 internal size standard (Applied Biosystems, Foster City, CA, USA). Alleles were scored by using Genotyper software (Applied Biosystems), and multilocus genotypes for each isolate were then generated by scoring length polymorphisms at the 6 microsatellite-containing loci. Microsatellite types were subsequently identified for newly typed isolates by comparing this novel dataset from China against previously genotyped isolates from Thailand (*10*).

### Genetic Data Analysis

Multilocus microsatellite types (MLMTs) were manipulated and analyzed by using the GenAlEx software add-in for Excel (*11*). The 86 Chinese isolates were coded into 2 populations according to host, human or rat, and were compared against 186 *P. marneffei* MLMTs from Thailand, also coded into 2 populations according to host. Isolates were further partitioned into 2 broad geographic regions, China or Thailand. Basic data exploration was undertaken to calculate allele and genotype frequencies and to calculate diversity statistics. The number of identical genotypes shared between hosts (human and rat) was then determined for each region by assessing which MLMTs were common to both host species. Subsequently, the genetic distances between the MLMT genotypes of *P. marneffei* from different hosts were calculated and visualized by using the neighbor-joining tree algorithm in GenAlEx (*11*).

The distribution of genetic variation across Southeast Asia between isolates of *P. marneffei* was estimated by performing an analysis of molecular variance (AMOVA) (*12*). AMOVA is a statistical technique that estimates the extent of genetic differentiation between individuals and populations directly from molecular data. The technique treats the raw molecular data as a pairwise matrix of genetic distances between all possible combinations of *P. marneffei* isolates, with submatrices corresponding to the different hierarchical data partitions (here, the genetic differences between *P. marneffei* infecting different host individuals, host species, and geographic regions). The data are then analyzed within a nested analysis of variance framework. Means squares are computed for each hierarchy of data, enabling significance testing between the following: 1) individual *P. marneffei* genotypes within hosts; 2) genotypes distributed between hosts (humans and bamboo rats); and 3) genotypes distributed between region (China and Thailand). Randomized distributions of the data are generated through random permutations, and the rejection of the null hypothesis (Ho = no significant component of variation occurs between the hierarchical divisions) then demonstrates the existence of population subdivision, either at the level of geography or host.

Subsequently, the presence of fine-scale geographic substructure within regions was determined by the use of Mantel tests. Mantel tests work by creating 2 pairwise matrices from each collection of isolates corresponding to 1) the pairwise genetic distances between isolates, and 2) the pairwise spatial distances between isolates in kilometers. The observed correlation between these genetic and geographic distances within China and for each host population (human and rat) were calculated and compared against 1,000 randomized datasets. The observed correlations were then considered significant (>0) if they exceeded 950 of the randomized datasets.

## Results

### Prevalence of *P. marneffei* in *R. pruinosus* Rats across Guangxi

Our survey demonstrated that 100% of the 43 adult *R. pruinosus* rats captured in Guangxi Province were positive for *P. marneffei* (Table 1). All the *P. marneffei*–positive *R. pruinosus* rats appeared healthy and, at necropsy, no visible pathologic changes were observed in any of the internal organs of the rats. The strains were most frequently isolated from lung, liver, and spleen tissues; however, no isolate was recovered from the embryonic tissue of pregnant rats (n = 15). This finding suggests that vertical transmission of infection within *R. pruinosis* rats does not occur. Forty isolates were obtained from human, including 36 patients who were positive for HIV. The strains were most frequently recovered from blood cultures (100%), followed by bone marrow aspirate (91%), skin biopsy specimens and pus of subcutaneous abscess (84%), lymph node biopsy specimens (34%), and bronchoalveolar lavage pellets (2.5%).

### MLMT Analyses of *P. marneffei* in Guangxi

A single PCR amplification product was observed for all 83 isolates at each locus. Because *P. marneffi* has a haploid genome, this finding suggests that none of the cultures were composed of a mixture of *P. marneffei* strains and that hosts were therefore infected with single-genotype infections. All 6 MLMTs were polymorphic; 65 alleles were found in the pooled *P. marneffei* populations (human and rat), ranging from 3 alleles for locus PM25 to 11 for locus PM5 (Table 2). The numbers of alleles, haploid gene diversity, and distribution of private alleles were significantly different between human- and rat-associated isolates of *P. marneffei* in Guangxi (Table 2). Human-associated *P. marneffei* isolates in Guangxi were more polymorphic (Table 3) and showed higher haploid genetic diversity (Table 3) and numbers of unique alleles (Table 3).

**Table 2:** Microsatellite loci scored in *Penicillium marneffei* isolates, Guangxi Province, People’s Republic of China

Populations sampled	Locus	No. samples	Mean no. alleles	Mean effective no. alleles
Human	*PM5*	40	11	7.767
	*PM6*	40	6	3.419
	*PM19*	40	8	6.838
	*PM22*	40	5	3.433
	*PM23*	40	5	1.810
	*PM25*	40	3	1.831
Rat	*PM5*	43	7	5.945
	*PM6*	43	4	3.332
	*PM19*	43	5	3.332
	*PM22*	43	5	2.039
	*PM23*	43	3	1.208
	*PM25*	43	3	2.158
Total		41.5	5.417	3.592

**Table 3:** Difference in allele numbers, haploid gene diversity, and distribution of private alleles between human- and rat-associated isolates of *Penicillium marneffei* in Guangxi Province, China, and Thailand

Location and population sampled	No. samples	No. haplotypes	Mean no. alleles (SE)	Mean effective no. alleles (SE)	Mean haploid genetic diversity (SE)	Mean no. private alleles (SE)
China						
Human	40	38	6.333 (1.145)	4.183 (1.036)	0.674 (0.076)	1.167 (0.543)
Rat	43	22	4.500 (0.619)	3.002 (0.677)	0.575 (0.094)	0.333 (0.211)
Thailand						
Human	163	51	6.667 (1.430)	1.840 (0.224)	0.410 (0.079)	1.167 (0.833)
Rat	23	11	3.833 (0.401)	1.967 (0.389)	0.404 (0.094)	0.000 (0.000)
Total	269	116	5.333 (0.524)	2.748 (0.364)	0.516 (0.047)	

**Figure 2:**
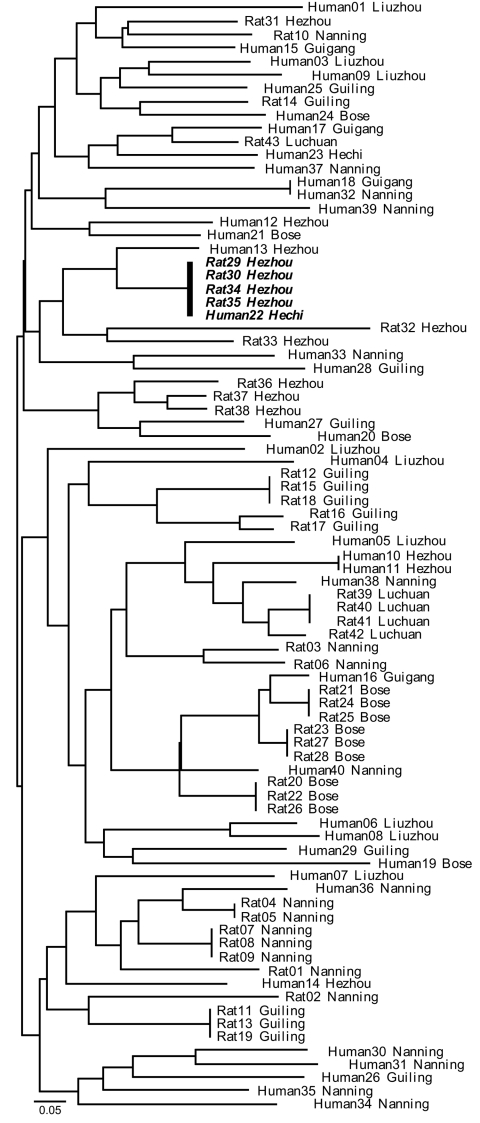
Neighbor-joining tree of the relationship between multilocus microsatellite type genotypes of human and bamboo rat associated *Penicillium marneffei* isolates, Guangxi Province, People’s Republic of China. Identical genotypes shared between humans and rats are in **boldface**. Scale bar indicates nucleotide substitutions per site.

MLMT analyses of the 83 isolates from Guangxi recovered 59 distinct multilocus microsatellite barcode types (MTs). A total of 38 MTs were found infecting humans, and 22 were infecting bamboo rats. A single MT haplotype was found to co-occur in both human and rats (Figure 2). The probability of observing infections with identical genotypes in different hosts by chance alone can be approximated as ≈5.46 × 10^6^; this occurrence is therefore statistically highly unlikely to occur, and we conclude that these 2 hosts were co-infected from a single clonally reproducing individual of *P. marneffei*. Although the bamboo rats in question were trapped in Hezhou, the human isolate was recovered from Hechi, 355 km distant; this observation could indicate either that the patient traveled to and acquired infection in the environs of Hezhou, or infectious *P. marneffei* conidia can disperse over that physical distance.

We then tested whether *P. marneffei* isolates from humans and rats represented genetically unique subpopulations of the total genetic diversity by using AMOVA (Table 4). Although isolates recovered from Thailand and China are clearly genetically isolated populations, only 2% of the total diversity was partitioned between host species within these regions. Within Guangxi, however, this minor component of diversity was significant (p = 0.008), which suggests that humans and bamboo rats in Guangxi are not infected by *P. marneffei* in a completely random manner and that some underlying genetic structure exists.

**Table 4:** Analysis of molecular variance among and within sampled populations of Penicillium marneffei*

Source	df	Estimated variance (%)	p value
Regions (China and Thailand)	1	0.974 (39)	>0.001
Among species (humans and rats)	2	0.059 (2)	>0.002
Within species	265	1.446 (58)	>0.001
Total	268	2.478 (100)	

*%, percentage of the total variance component across the specified hierarchy; p, probability that the observed variance is greater than that expected for the null hypothesis *H*o, generated by 999 random permutations of the data.

To test whether this component of variation between humans and bamboo rats was related to spatial factors, we tested the extent of correlation between geographic distance and genetic distance across 3 components of *P. marneffei* diversity in Guangxi by using Mantel tests for the following data partitions: 1) human and bamboo rat isolates together; 2) human isolates alone; and 3) bamboo rat isolates alone. Combined human and bamboo rat data showed a significant spatial component to the distribution of genetic diversity across Guangxi (p = 0.001). However, this effect is not observed for isolates collected from humans (p = 0.323) but is observed for isolates collected from bamboo rats (p = 0.001). This result shows that the spatial signal in the dataset is attributable to the rodent-associated isolates.

The finding that rodent isolates are more spatially structured than human-associated isolates is confirmed by assessing the proportion of identical genotypes that are to be found infecting either human or rodent hosts within the different sample sites in Guangxi (Figure 2). This analysis showed that although 9 MLMT haplotypes infected >1 bamboo rat within a sample site, only a single MLMT haplotype was shared among human patients within 1 sample site (Hezhou). That no MTs were shared by bamboo rats between trapping sites is strong evidence that *P. marneffei* genotypes are spatially patchy within Guangi Province. Furthermore, although no MLMT haplotypes were shared between bamboo rats from different sample sites, 2 MLMT haplotypes (Human18, Guigang and Human32, Nanning; Human22, Hechi and Rat29, 30, 34, and 35, Hezhou) were shared between humans or bamboo rats from different sample sites. Together, these data show that humans are exposed to a greater diversity of *P. marneffei* genotypes relative to the bamboo rats, despite occupying a similar geographic region.

## Discussion

The body of current evidence suggests that bamboo rats play a key role in the life cycle of *P. marneffei.* Efforts to detect live *P. marneffei* in the soil environment have generally failed, although *P. marneffei* DNA has been detected in soils in Thailand that have a known association with animals, such as elephants (*13*). *P. marneffei* is consistently and reproducibly isolated from several species of bamboo rat across its known range and, within China, Li et al. (*14*) and Deng et al. (*15*) have shown that infection in these rodents exhibits a high prevalence of infection in *R. pruinosus*. In this study, we used a sample of 43 animals from 5 sample sites across a wide geographic region spanning 500 km to show that infection is prevalent at 100% in all sampled populations. Therefore, as in Thailand and India, *P. marneffei* in China appears to be strongly associated with bamboo rats (*7**,**15**–**17*), underscoring the likely role of these rodents in the life cyle of this mycosis.

To address this question, we ascertained whether *P. marneffei* isolates from bamboo rats in China were the same as, or different from, those infecting humans across this region. Our use of molecular genotyping clearly showed little difference in allele frequencies between these 2 host-associated populations of *P. marneffei*, and in 1 case direct sharing of a common genotype was observed, showing that human and bamboo rat infections in China are highly similar. This finding parallels reports from Thailand and India (*7**,**10*), where little difference between human and rodent isolates was observed. Taken together, these observations strongly suggest that all bamboo rat associated isolates are potentially able to cause infections in humans.

However, as was observed in Thailand (*10*), Chinese bamboo rat isolates represent a more spatially heterogeneous population of *P. marneffei* compared with human infections and exhibit lower gene-diversity indices and lower relatedness across large spatial regions and higher clonality (as shown by identical genotypes) within a sampled site. From a strictly genetic standpoint, the spatially clustered clonal isolates infecting the bamboo rat populations relative to the higher diversity, nonclustered isolates observed in human infections could be explained by 2 hypotheses: The first hypothesis is that humans travel more widely than bamboo rats, and therefore sample a greater number of *P. marneffei*–infected environments. The second hypothesis is that bamboo rat and human infection reflect different aspects of the sylvatic reservoir of *P. marneffei*. In this second hypothesis, rodents become infected by spores that are locally produced, reflecting the innate spatial clustering of sylvatic spore dispersal. However, some aerosolized conidia may be dispersed beyond forests, thus generating a relatively more homogeneous, but less dense, population of infectious conidia that are able to infect persons with HIV outside of the naturally occurring ecologic niche for *P. marneffei*.

## Conclusions

We were not able to discriminate between our 2 hypotheses; however, we did identify the type of questions that we need to be asking. First, we need to establish whether infected humans are widely exposed by traveling within potentially *P. marneffei*– and bamboo rat–associated habitats: the answer to this is likely to be no because 1) there is no recognized epidemiologic association between infection and travel to bamboo rat habitats (*18*); and 2) the infected population tends to be urbanized sex workers and drug users who have HIV. In this case, if humans are acquiring their infections within urban environments by inhaling aerosolized conidia, then conidia should be detectable by the use of high-throughput air sampling and molecular-probe detection of *P. marneffei*. In support of this technique, a broad-scale sampling of soils in Thailand that used quantitative PCR (*13*) showed that *P. marneffei* could be patchily detected in animal-associated soils; such technology could be readily adapted to be used with air-sampling technology.

Finally, if bamboo rats are a major amplification reservoir for *P. marneffei* by the pathogen being sequestered within bamboo rats and becoming transmissible upon host death, then domestic or pest animal hosts may be involved. However, we know little about the host range of *P. marneffei*. Therefore, given the widespread increase in the prevalence of this infection across Southeast Asia, we assert that there is a pressing need to revisit the epidemiology of this highly enigmatic infection and that the occurrence of realized and potential amplifiers of human infection need to be reassessed.

## References

[1] Vanittanakom N, Cooper CR Jr, Fisher MC, Siristhanthana T. *Penicillium marneffei* infection and recent advances in the epidemiology and molecular biology aspects. Clin Microbiol Rev. 2006;19:95–110. 10.1128/CMR.19.1.95-110.2006 16418525 PMC1360277

[2] Wong KH, Lee SS, Chan KC, Choi T. Redefining AIDS: case exemplified by *Penicillium marneffei* infection in HIV-infected people in Hong Kong. Int J STD AIDS. 1998;9:555–6. 9764944

[3] Supparatpinyo K, Khamwan C, Baosoung V, Nelson KE, Siristhanthana T. Disseminated *Penicillium marneffei* infection in Southeast Asia. Lancet. 1994;344:110–3. 10.1016/S0140-6736(94)91287-4 7912350

[4] Zhiyong Z, Mei K, Yanbin L. Disseminated *Penicillium marneffei* infection with fungemia and endobronchial disease in an AIDS patient in China. Med Princ Pract. 2006;15:235–7. 10.1159/000092189 16651843

[5] Capponi M, Segretain G, Sureau P. Penicillosis from *Rhizomys sinensis.* Bull Soc Pathol Exot. 1956;49:418–21. 13364636

[6] Wieden MA, Saubolle MA. The histopathology of coccidioidomycosis. In: Einstein HE, Catanzaro A, editors. Coccidioidomycosis: proceedings of the 5th International Conference on Coccidioidomycosis, Stanford University, 24–27 August 1994. Bethesda (MD): National Foundation for Infectious Diseases; 1996. p. 12–7.

[7] Gugnani H, Fisher MC, Paliwal-Johsi A, Vanittanakom N, Singh I, Yadav PS. Role of *Cannomys badius* as a natural animal host of *Penicillium marneffei* in India. J Clin Microbiol. 2004;42:5070–5. 10.1128/JCM.42.11.5070-5075.2004 15528698 PMC525236

[8] Cao C, Liu W, Li R. *Penicillium marneffei* SKN7, a novel gene, could complement the hypersensitivity of *S. cerevisiae* skn7 Disruptant strain to oxidative stress. Mycopathologia. 2009;168:23–30. 10.1007/s11046-009-9192-x 19294341

[9] Fisher MC, Aanensen D, de Hoog S, Vanittanakom N. Multilocus microsatellite typing system for *Penicillium marneffei* reveals spatially structured populations. J Clin Microbiol. 2004;42:5065–9. 10.1128/JCM.42.11.5065-5069.2004 15528697 PMC525240

[10] Fisher MC, Hanage WP, de Hoog S, Johnson E, Smith MD, White NJ. Low effective dispersal of asexual genotypes in heterogeneous landscapes by the endemic pathogen *Penicillium marneffei.* PLoS Pathog. 2005;1:e20. 10.1371/journal.ppat.0010020 16254598 PMC1266309

[11] Peakall R, Smouse PE. GenAlEx 6: genetic analysis in Excel. Population genetic software for teaching and research. Mol Ecol Notes. 2006;6:288–95. 10.1111/j.1471-8286.2005.01155.x PMC3463245 22820204

[12] Excoffier L, Smouse PE, Quattro JM. Analysis of molecular variance inferred from metric distances among DNA haplotypes: application to human mitochondrial DNA restriction data. Genetics. 1992;131:479–91. 1644282 10.1093/genetics/131.2.479 PMC1205020

[13] Pryce-Miller E, Aanensen D, Vanittanakom N, Fisher MC. Environmental detection of *Penicillium marneffei* and growth in soil microcosms in competition with *Talaromyces stipitatus.* Fungal Ecol. 2008;1:49–56. 10.1016/j.funeco.2008.02.002

[14] Li JC, Pan LQ, Wu SX. Mycologic investigation on *Rhizomys pruinous senex* in Guangxi as natural carrier with *Penicillium marneffei.* Chin Med J (Engl). 1989;102:477–85. 2512073

[15] Deng ZL, Yun M, Ajello L. Human penicilliosis marneffei and its relation to the bamboo rat (*Rhizomys pruinosus*). J Med Vet Mycol. 1986;24:383–9. 10.1080/02681218680000581 3783360

[16] Ajello L, Padhye AA, Sukroongreung S, Nilakul CH, Tantimavanic S. Occurrence of *Penicillium marneffei* infections among wild bamboo rats in Thailand. Mycopathologia. 1995;131:1–8. 10.1007/BF01103897 8532047

[17] Chariyalertsak S, Sirisanthana T, Supparatpinyo K, Nelson KE. Seasonal variation of disseminated *Penicillium marneffei* infections in northern Thailand: a clue to the reservoir? J Infect Dis. 1996;173:1490–3. 10.1093/infdis/173.6.1490 8648227

[18] Chariyalertsak S, Sirisanthana T, Supparatpinyo K, Praparattanapan J, Nelson KE. Case–control study of risk factors for *Penicillium marneffei* infection in human immunodeficiency virus–infected patients in northern Thailand. Clin Infect Dis. 1997;24:1080–6. 10.1086/513649 9195061

